# Hypoglycin A Content in Blood and Urine Discriminates Horses with Atypical Myopathy from Clinically Normal Horses Grazing on the Same Pasture

**DOI:** 10.1371/journal.pone.0136785

**Published:** 2015-09-17

**Authors:** M. Bochnia, J. Ziegler, J. Sander, A. Uhlig, S. Schaefer, S. Vollstedt, M. Glatter, S. Abel, S. Recknagel, G. F. Schusser, M. Wensch-Dorendorf, A. Zeyner

**Affiliations:** 1 Institute of Agricultural and Nutritional Sciences Martin Luther University Halle-Wittenberg, Halle (Saale), Germany; 2 Department of Molecular Signal Processing, Leibniz Institute of Plant Biochemistry, Halle (Saale), Germany; 3 Screening Labor, Hanover, Germany; 4 Department of Large Animals Medicine, Faculty of Veterinary Medicine, Leipzig, Germany; 5 Traditional Chinese Equine Medicine, Bokholt-Hanredder, Germany; University of Minnesota, UNITED STATES

## Abstract

Hypoglycin A (HGA) in seeds of *Acer spp*. is suspected to cause seasonal pasture myopathy in North America and equine atypical myopathy (AM) in Europe, fatal diseases in horses on pasture. In previous studies, this suspicion was substantiated by the correlation of seed HGA content with the concentrations of toxic metabolites in urine and serum (MCPA-conjugates) of affected horses. However, seed sampling was conducted after rather than during an outbreak of the disease. The aim of this study was to further confirm the causality between HGA occurrence and disease outbreak by seed sampling during an outbreak and the determination of i) HGA in seeds and of ii) HGA and MCPA-conjugates in urine and serum of diseased horses. Furthermore, cograzing healthy horses, which were present on AM affected pastures, were also investigated. AM-pastures in Germany were visited to identify seeds of *Acer pseudoplatanus* and serum (n = 8) as well as urine (n = 6) from a total of 16 diseased horses were analyzed for amino acid composition by LC-ESI-MS/MS, with a special focus on the content of HGA. Additionally, the content of its toxic metabolite was measured in its conjugated form in body fluids (UPLC-MS/MS). The seeds contained 1.7–319.8 μg HGA/g seed. The content of HGA in serum of affected horses ranged from 387.8–8493.8 μg/L (controls < 10 μg/L), and in urine from 143.8–926.4 μg/L (controls < 10 μg/L), respectively. Healthy cograzing horses on AM-pastures showed higher serum (108.8 ± 83.76 μg/L) and urine concentrations (26.9 ± 7.39 μg/L) compared to control horses, but lower concentrations compared to diseased horses. The range of MCPA-carnitine and creatinine concentrations found in diseased horses in serum and urine were 0.17–0.65 mmol/L (controls < 0.01), and 0.34–2.05 μmol/mmoL (controls < 0.001), respectively. MCPA-glycine levels in urine of cograzing horses were higher compared to controls. Thus, the causal link between HGA intoxication and disease outbreak could be further substantiated, and the early detection of HGA in cograzing horses, which are clinically normal, might be a promising step in prophylaxis.

## Introduction

In horses on pasture, atypical myopathy (AM) and the similar disorder called seasonal pasture myopathy (SPM), are highly fatal forms of non exertional rhabdomyolysis occurring mainly in autumn but also less frequently in the subsequent spring [1–4]. Particular, season-dependent pasture characteristics seem to promote the incidence of AM or SPM [4], [5]. The first cases of AM were reported in 1939 in the North of Wales [6] and since the 1980s, also in various continental European countries [7–10]. The similar disease, SPM, is mainly described in North America [11], [12]. The first continental European outbreak occurred in Northern Germany in 1995 [12], whereby nearly all of the AM-affected horses died (111/115). However, the quantity of affected horses on the respective pasture is not uniform worldwide. In Europe large outbreaks with many affected horses on the same pasture are described, whereas in North America only a few horses with the same conditions develop the disease [2], [11], [13], [14]. Thus, cograzing horses exhibiting no disease symptoms have not been studied alongside affected horses.

In addition to the presence of *Acer spp*. *(Acer negundo*, *Acer pseudoplatanus)*, other pasture-related risk factors exist [14], [15]. Gusts of wind have been reported to coincide with an outbreak or were discussed to be a forerunner, respectively [4], [11], [16]. Predisposed horses for AM or SPM have been found to be predominantly young males (< 3 years; [14]) that were kept on poorly maintained pastures (often more than 12 hours a day) without supplementary feeding. Previous studies revealed increased prevalence of AM in females, but this is probably due to the more frequent exposure to pastures compared to males [4]. The prevalence in young horses might be due to the fact, that horses until the age of three years spend more time on pastures until horse owners start to work with them [4]. Several hypotheses about the biochemistry underlying disease development have been communicated in the past, but the discovery of the disruption of fatty acid ß-oxidation and amino acid metabolism by an acquired enzymatic deficiency of multiple acyl-CoA dehydrogenases (MADD) was a major step in diagnostic investigation [17]. As a consequence, there is excessive myofiber lipid storage and an abnormal production of blood acylcarnitines and urine organic acids [2], [17]. MADD is also described in humans. Clinical signs of inherited forms in humans range from a fatal neonatal condition to an adult onset mild lipid storage myopathy [18]. After ingestion of unripe Jamaican ackee fruit (*Blighia sapida*), whose seeds contain a nonproteinogenic amino acid called hypoglycin A (HGA, L-α-amino-methylenecyclopropylpropionicacid), an acquired form of MADD in humans can develop, with hypoglycaemia, continuing vomiting, and possible fatal outcome. HGA is metabolized to MCPA (methylencyclopropylacetic acid) and binds to CoA and is consequently a potent inhibitor of Acyl-CoA-dehydrogenases [19]-[20]. In horses with MADD, clinical manifestations include muscular weakness, stiffness, trembling, sweating, and myoglobinuria [1], [17]. Very indicative signs are severe acute myonecrosis of respiratory and postural muscles and less frequently the myocardium [4], [21], [22]. The serum activities of the creatine kinase (CK) and lactate dehydrogenase (LDH) are extremely elevated. The majority of horses developed recumbency and respiratory difficulties. In 75% of all cases the outcome was lethal [4], [7], [12], [15], [23].

Chase et al. [24] determined HGA concentrations in unripe ackee fruit components as high as 939, 711, and 41.6 mg/100 g of seed, aril, and husk components, respectively. Analysis of the ripe fruit components showed a remarkable decrease by about 70% in seeds. A current study conducted by Bowen-Forbes et al. [25] across two different harvest seasons of ackee fruits shows a strong inverse relationship between HGA and hypoglycin B (HGB), a dipeptide of glutamic acid and hypoglycin, which is also toxic. HGA decreased from 80 mg/100 g in green seeds to 14 mg/100 g in ripened seeds whereas HGB increased from 16 mg/100 g to 118 mg/100 g. The authors reasoned that the rise in HGB content represents a possible detoxification mechanism of the fruit.

HGA was also found in seeds of box elder trees (*Acer negundo*, *Sapindaceae*), which belongs to the same taxonomic plant family as *Blighia sapida*. Interestingly, box elder trees were present on pastures in North America with an increased incidence of SPM in horses [16]. Additionally toxic HGA metabolites were found in urine (MCPA-glycine) and in serum (MCPA-carnitine) of affected horses suggesting the ingestion of seeds of *Acer spp*. [16]. In Europe another *Acer spp*. called *Acer pseudoplatanus* (Sycamore maple) was identified on all pastures with horses affected with AM [26]. Subsequent (> 1 year later) analysis of HGA-content in Sycamore maple seeds of AM-affected farms could detect a HGA content (3.6–252.9 μg/seed [27]; 0.74 and 7.2 mg/g seed [28]) similar to previous studies (3.0–160.0 μg/seed; [16]). In addition to the ackee fruit (*Blighia sapida*) hypoglycin A or hypoglycin-like compounds were also detected in different chestnuts by Bressler et al. [29], for example *Aesculus parviflora*, *Aesculus glabra* as well as in many other species. All studies noticed a high variation of HGA content in different plants according to the level of maturity. Unger et al. [27] also showed a high variation of HGA content in the seeds of *Acer pseudoplatanus*, but a direct link between maturity and measured HGA concentrations is missing. In order to provide toxicological evidence of ackee, Fincham [30] measured HGA in blood and plasma of human patients. However, the detection limit was too high (1.4 mg/L) because HGA is already toxic at lower concentrations. A more sensitive HGA detection method to improve the prevention of intoxication of ackee or maple poisoning was presented approximately 40 years later [28] with a detection limit for HGA of 0.35 μg/L blood. The first quantitative determination of HGA in serum samples of two AM affected horses showed a HGA content of 446.9 and 87.8 μg/L [28]. However, there was no proof between the consumption of HGA containing maple seeds and the disease.

We hypothesized that the outbreak of AM in horses on pastures in Germany was caused by the ingestion of seeds of sycamore maple tree that contained high amounts of HGA. Toxic HGA and its metabolites (MCPA-conjugates) could be identified in the serum and urine of the affected horses. Cograzing healthy horses coexisting on affected pastures were also tested to determine the concentration of HGA in order to establish a link between HGA levels in serum and urine and their clinical disease.

## Material and Methods

### Case selection

In autumn of 2013 and 2014, 16 horses (7 Warmbloods, 4 Haflinger, 5 ponies; comprising 7 geldings, 2 stallions, 7 mares) were studied, which were turned out on pastures in Germany and exhibited acute clinical signs of muscle pain and weakness. The farm veterinarians conducted the initial treatment and diagnosed AM based on the clinical signs and clinicopathological results. Five horses were admitted to the equine clinic of the Department of Large Animals Medicine, Faculty of Veterinary Medicine Leipzig, Germany, for intensive care where they died or were euthanized. In addition to the preliminary report, clinical signs included the sudden onset of indicator signs of acute rhabdomyolysis (e.g. myoglobinuria, stiffness, trembling, sweating), weakness, recumbency and depression, with rapid progression of signs or even unexpected death. Of the investigated cases 15 horses died and one survived. Eight serum samples and 6 ante-mortem urine samples were collected from these affected horses during the disease. Additionally 12 horses that were clinically normal and cograzing pastures with AM cases were included in the study.

### Ethics statement

Animal Care and Use Committee approval was not obtained for this study because no animals were handled specifically for this experiment. Blood and urine samples were collected by qualified veterinarians through their routine practice, in the framework of official programs aimed at the diagnosis and a possible treatment. Total duration of the blood sampling procedure did not exceed 1 min, and non-painful sampling was confirmed by the absence of any retreat behavior of the horses during the procedure. Urine samples were collected as free-catch urine. The detection of the contents of HGA and MCPA-conjugates was conducted with the owner's consent. An experienced botanist and a veterinarian executed the botanical rating and collection of seed samples on affected pastures.

Therefore, the legal restrictions do not apply, as they are waived in the case of non-experimental procedures and routine veterinary practices with livestock species (no laboratory animals).

### Botanical inspection and owner interview

**Fig 1:**
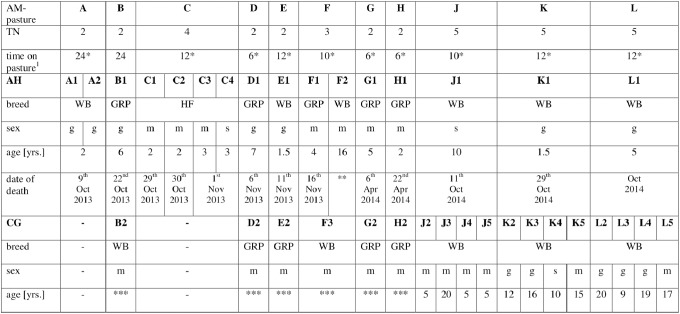
Specific characteristics of AM-affected and cograzing horses dedicated to the individual AM-pasture. TN = total number of horses on individual pasture; ^1^ in hrs/d, *additional food stuffs (hay, concentrate); AH = affected horses, CG = cograzing horses: the denomination is made up of the letter of pasture (A-L) and the number of affected or cograzing horses on this pasture; WB = Warmblood, GRP = German Riding Pony, HF = haflinger; g = gelding, m = male, s = stallion; **survived, affect date 17^th^ Nov 2013, ***not available.

The AM and cograzing horses originated from 11 different pastures (pasture A-L, Fig 1). Nine affected pastures were visited directly after the occurrence of AM or at latest 14 days after the outbreak to collect seed samples. An experienced botanist and a veterinarian, with the owner´s consent, conducted the botanical rating. Digital images were taken and available trees and plants were obtained and cataloged. Samples of grass, leaves and seeds were collected. The focus was directed towards *Acer spp*., especially sycamore maple trees (*Acer pseudoplatanus*) to determine the presence of the seeds and the availability of those to the horses. Individual seed sampling in 2013 from soil was conducted according to the protocol of Valberg et al. [16] (pasture B, C, E, F). In 2014 the sampling method was adapted according to the protocol of Unger et al. [27] with pooled samples from the trees (pasture G, H, J, K, L). Pasture A and D were not visited, because it was too late for seed sampling (information about these cases more than 4 weeks later). Additionally the owners were asked about the development of AM (first admission to the pasture, date of first clinical signs, composition of the herd, hours on pasture, additional food stuffs).

### MCPA-conjugates analysis

Samples of affected horses obtained immediately before demise and within 3 days of the onset of AM disease were available for analysis. Acylconjugates concentrations were analyzed by ultra performance liquid chromatography tandem mass spectrometry (UPLC-MS/MS) to identify MCPA-carnitine and-glycine in equine serum and urine in a special screening laboratory in Hannover, Germany [31]. Samples of cograzing horses were tested as well. Owners gave permission for measuring MCPA-conjugates in available body fluids. Serum samples of 14 horses without any symptoms of AM and without contact to seeds of *Acer spp*. on pastures were used as controls. In these cases blood was drawn as part of routine screening or in the diagnostic work up of cases unrelated to AM. Five urine samples of horses were collected as free-catch urine from healthy horses as negative controls. All samples were frozen at -20°C until they were sent to the laboratory for analysis.

### Hypoglycin A analysis

Authentic *S*-Hypoglycin A was from Toronto Research Chemicals (Toronto, ON). Plant tissue (between 100 and 300 mg) were ground to a fine powder with a mortar and pestle under liquid nitrogen and subsequently further homogenized with a mixer mill MM301 (Retsch, Haan, Germany) at a frequency of 25/s for 50 s with a single 5 mm diameter steel bead in a 2 ml Eppendorf cup. The resulting powder was extracted with 750 μl 70% (v/v) methanol containing 10 nmol of norvaline with vigorous shaking and the slurry was centrifuged twice at 10,000 g for 5 min.

HGA was analyzed by LC-ESI-MS/MS and quantified as its Fmoc-derivative according to the protocol for amino acid determination published by Ziegler et al. [32] using 25 μl of plant extract or 5 μl of serum and urine samples, respectively. HGA contents in affected and cograzing horses were determined. Owners gave permission for measuring HGA in available body fluids. Serum (n = 5) and urine (n = 4) samples of horses without any symptoms and without contact to *Acer pseudoplatanus* were used as controls. Blood in these cases was drawn as part of routine screening or in the diagnostic work up of cases unrelated to AM. Four urine samples of horses were collected as free-catch urine from healthy horses as negative controls. All samples were frozen at -20°C until analysis.

MS parameters describing the MRMs for the HGA are shown in the Supporting information (S1 Table).

### Hypoglycin A calculation in mg HGA/horse

For calculation of HGA concentration in mg HGA/horse to reveal the maximal tolerated dosage (MTD) the following parameters were used:

1. measured HGA concentration in serum (μg/L) of affected, cograzing and control horses
2. estimated total blood volumes ([33], 101 ml/kg bwt), which was calculated with the bwt.

$$\begin{array}{l} \text{For example}:\;500\;kg\;bwt\;*\;101ml\;blood/kg\;bwt\;=\;50.50\;L\;blood\\ \;50.50\;L\;blood\;*\;detected\;concentration\;\mu g\;HGA/L\;=\;\mu g\;HGA/horse\\ \;\left(\mu g\;HGA/horse\right)\;/\;1000\;=\;mg\;HGA/horse \end{array}$$

### Statistical analysis

For the subsequent multiple comparison post hoc test a SAS based macro according to the protocol of Elliott and Hynan [34] was used to reveal differences between the concentrations of HGA in serum and urine of affected and cograzing horses in comparison to the controls. The macro takes into account different group sizes according to Dunn’s multiple comparison procedure [34]. Since the data from HGA in urine and serum are not normally distributed, ANOVA could not be used. However, since the data are independent and continuous, and the null hypothesis of Levene‘s Test to test the homogeneity of variance could not be rejected, the nonparametric Kruskal-Wallis Test to test for difference among groups was applied. The null hypothesis of the Kruskal-Wallis Test was rejected for both traits, i.e. that at least 2 groups are different.

## Results

### Case history, outcome of owner interview and clinical findings

**Fig 2:**
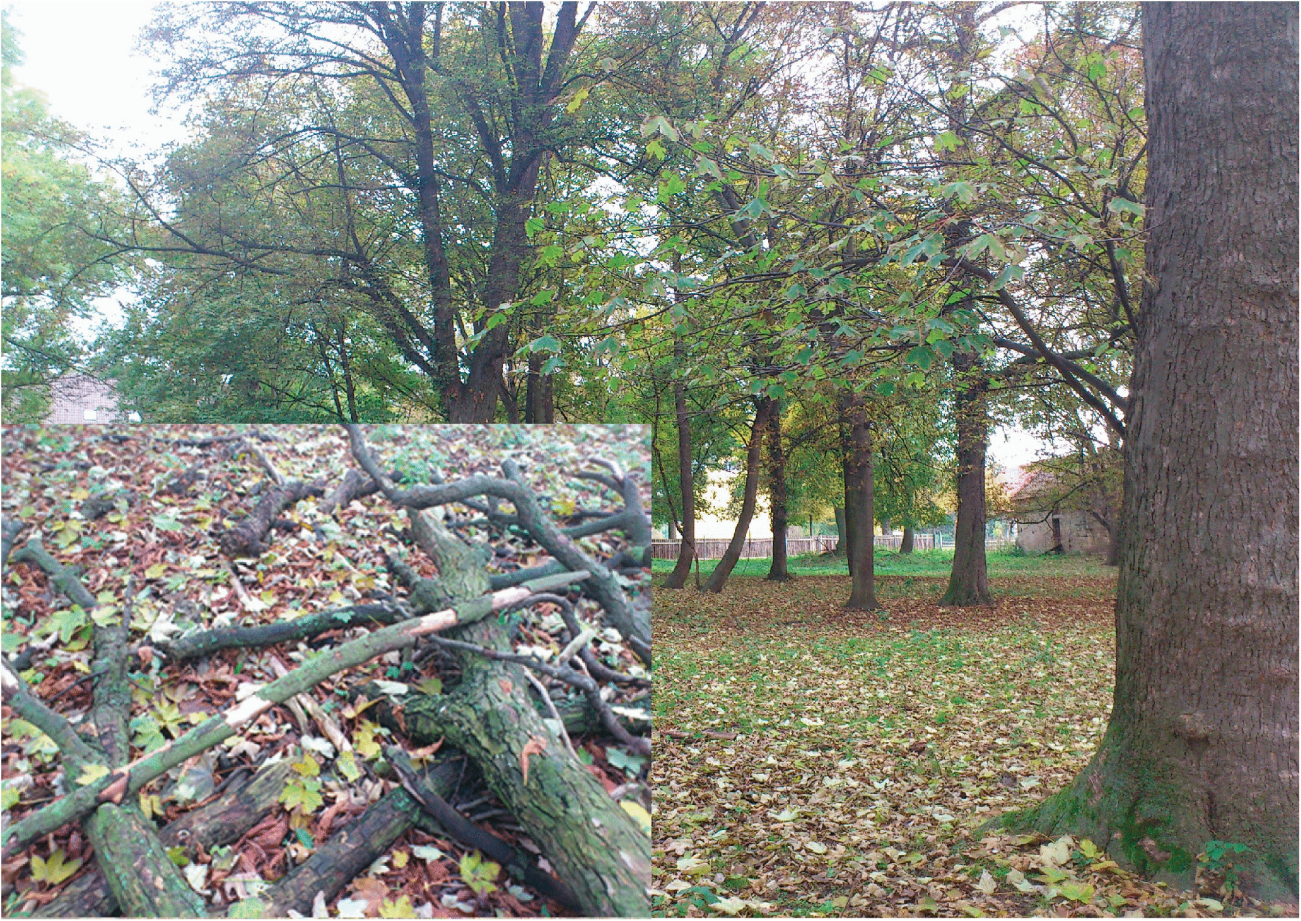
Pasture A: a typical AM-pasture in autumn with many trees and very sparse grassland.

The affected horses comprised 3 different breeds (Warmblood, Haflinger, German Riding Pony) and included 7 geldings, 2 stallions and 7 mares aged from 1.5–16 years (Fig 1). All affected horses had a good body condition (ranged from 5.0–5.5/9) and the turnout time onto the pasture was variable between the cases (6–24 hrs/d, Fig 1). Trees were present on or around all pastures and horses had access to dead wood and leaves. Pastures were poorly maintained and the grassland was in most cases very sparse, the turf was often damaged with soil showing through and a lot of dead leaves (Fig 2).

All horses (except horse No. B1) got additional feed such as hay and cereals (oat grains) or a mixed feed. Hay was offered ad libitum at stable with free access to the pastures (personal communication with the owner) or was additionally provided at pasture with a hayrack. Cereals or mixed feeds were portioned once or twice a day. All had access to a salt or mineral block and all were routinely dewormed. A total of three horses were used for riding (B1, J1; 2–3 time per week) or driving (D1; 1–2 times per week). The other horses had not been exercised, mainly because of their age (< 3 years).

All horses exhibited typical clinical symptoms of AM, as it was described in several previous studies [1–5], [13–16]. Interestingly the horses C1, C2, C3 and C4 from the same pasture developed AM consecutively within 48 hours, although access to pasture was constrained after the first horse showed the onset of disease. Hence, although the seeds containing toxic amino acid HGA were no longer available, the horses succumbed to disease within two days (Fig 1). Horse C4 was the last horse from this pasture becoming ill and the blood sample showed a moderate increase in CK. However, this horse was euthanized at a very early stage of the disease while it was still standing. One horse died within 6 hours (F1), owners found the horse dead on pasture. The second and oldest (16 years) horse on this pasture (F2) survived, and the concentrations of CK and LDH were only moderately increased. After giving the horse intensive care (treatment included intravenous administration of fluids 70–100 mL/kg bwt/day, flunixin-meglumin over 7 days) a paralysis of the tongue and a slightly lameness was observed, but this was reversible after 4–6 weeks. Available blood samples from all horses showed a marked increase in CK (range 6,154–1,215,987 U/L, normal: Warmblood 146–346 U/L, Haflinger and Pony 181–652 U/L [35]) and LDH (range 816–43,802 U/L, normal: Warmblood 223–536 U/L, Haflinger and Pony 293–822 U/L [35]).

### MCPA-carnitine and acylcarnitines in serum and urine

Serum and urine acylcarnitines profiles resembled that of MADD in all analysed samples (Tables 1 and 2). Concentrations of medium chain acyl conjugates in serum were at least ten times higher compared to controls, whereas urine samples of affected horses exhibited a 1,00–1,000 times higher level compared to control samples. High MCPA-carnitine was found in all sera of severely diseased horses, but MCPA-glycine was present at very low concentrations. However, in urine the MCPA-glycine concentrations were high in all samples obtained from diseased animals. In all cases, high concentrations of acylcarnitines (e.g. Butyryl-, Isovaleryl-, Valeryl-carnitine) and MCPA-conjugates (MCPA-carnitine and–glycine) in serum corresponded to elevated levels in urine. Especially horse C4 showed a moderate increase beyond all parameters. However, this horse was euthanized at a very early stage of the disease and therefore detected concentrations are much lower in comparison to the other affected horses.

**Table 1:** Measured concentrations of MCPA-conjugates and acylcarnitines (C4:1 –C10:1) in serum of affected and cograzing horses compared to controls determined by UPLC-MS/MS.

Acylcarnitine and MCPA-conjugates [μmol/L]	AH						CG	Controls
	A1	A2	B1	C4	D1	E1		
MCPA-glycin	0.023	0.010	0.014	0.016	0.008	0.024	*)	*)
Valerylglycin	1.74	1.7	1.86	0.88	6.32	3.75	*)	*)
Hexanoylglycin	2.72	3.7	3.95	2.23	4.5	5.83	*)	*)
Isobutyryl-carnitine	13.00	15.88	3.57	3.0	1.75	6.73	0.63±0.09	0.54
Butyryl-carnitine	100.64	121.87	60.8	43.95	37.23	58.51	0.78±0.18	0.67
Isovaleryl-carnitine	43.1	44.9	24.32	12.38	16.46	25.78	0.22±0.04	0.18
Valeryl-carnitine	1.57	2.24	1.07	0.39	0.44	0.94	0.01±0	*)
MCPA-carnitine	0.459	0.652	0.508	0.166	0.320	0.459	0.002±0.001	*)
Hexanoyl-carnitine	29.24	39.9	34.4	11.62	8.87	25.08	0.29±0.09	0.24
Octanoyl-carnitine	2.01	2.80	2.51	0.68	0.88	2.22	0.04±0.09	*)
Decenoyl-carnitine	0.47	0.84	0.4	0.11	0.16	0.42	0.02±0	*)

AH = affected horses

CG = cograzing horses (means±sd)

*) below limit of detection (< 0.001μmol/L).

**Table 2:** Measured concentrations of MCPA-conjugates and acylcarnitines (C4:1 –C10:1) in urine of affected and cograzing horses compared to controls determined by UPLC-MS/MS.

Acylcarnitine and MCPA-conjugates [μmol/mmoL creatinine]	AH				CG	Controls
	A1	B1	D1	E1		
MCPA-glycin	0.28	1.23	1.97	1.03	0.015±0.012	*)
Valerylglycin	72.68	208.10	286.80	159.75	3.99±1.28	0.50
Hexanoylglycin	133.32	256.81	192.85	305.83	0.29±0.09	*)
Isobutyryl-carnitine	55.74	50.06	57.46	47.21	0.78±0.36	0.31
Butyryl-carnitine	105.72	83.07	59.02	207.92	0.03±0.01	0.01
Isovaleryl-carnitine	34.1	23.39	29.23	75.35	0.01±0	0.01
Valeryl-carnitine	7.8	11.72	14.74	9.31	*)	*)
MCPA-carnitine	0.34	2.05	1.98	0.92	*)	*)
Hexanoyl-carnitine	35.77	55.20	38.22	78.87	*)	*)
Octanoyl-carnitine	7.21	15.56	11.46	17.3	*)	*)
Decenoyl-carnitine	4.56	14.87	13.04	11.37	*)	*)

AH = affected horses

CG = cograzing horses (means±sd)

*) below limit of detection (< 0.0001 μmol/mmoL creatinine).

In healthy, cograzing horses increased concentrations of the glycine-fraction (Valerylglycine, Hexanoylglycine, MCPA-glycine) in urine were observed. The levels of Valeryl-carnitine in body fluids were higher than the concentrations in controls, but much lower compared to the concentrations of affected horses. MCPA-carnitine in serum was detectable only in very low concentrations.

### Hypoglycin A assay in seeds and bodyfluids

**Fig 3:**
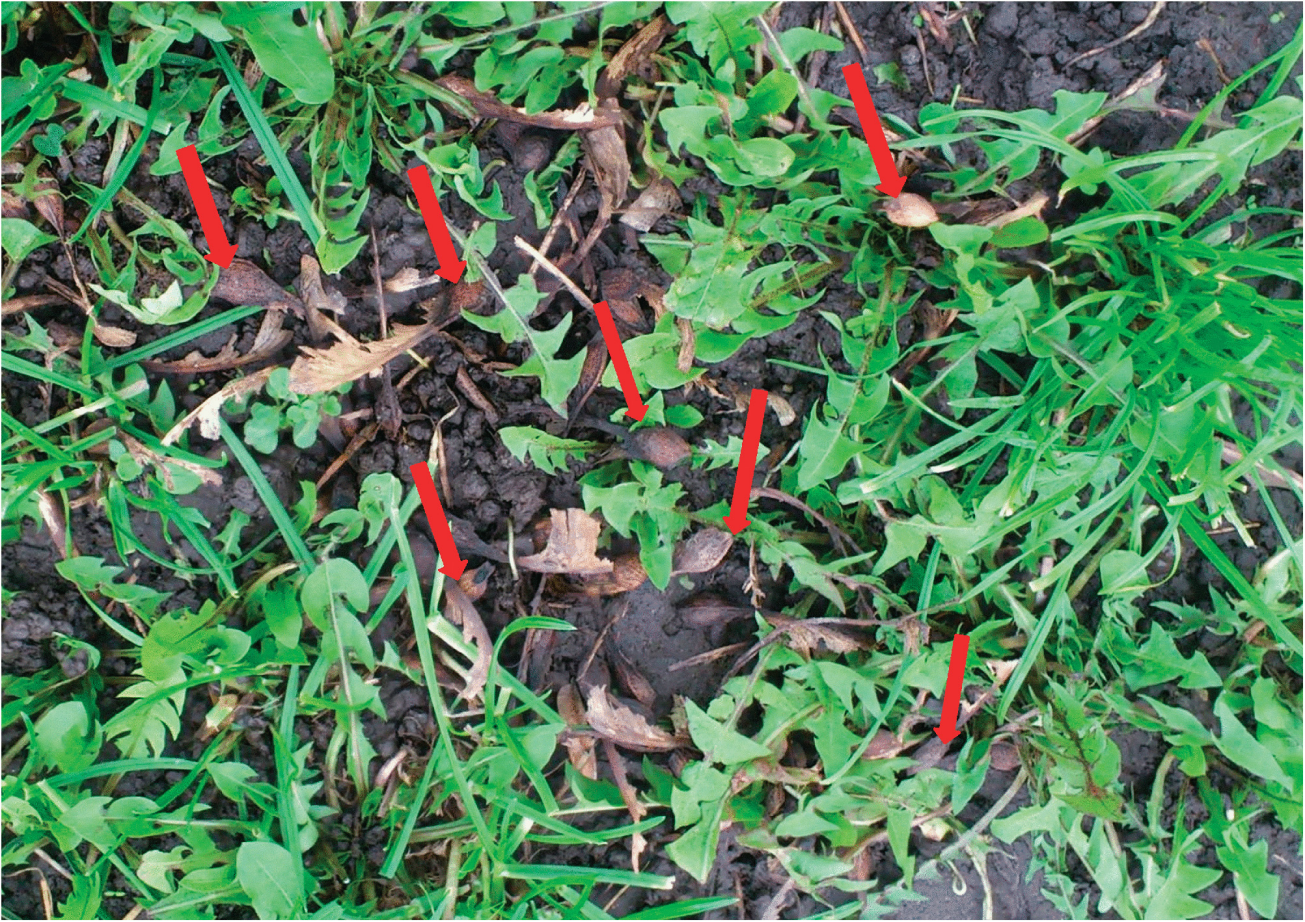
Pasture B. Numerous seeds found on pasture where the closest sycamore maple tree was 50 meters away from the pasture.

There was a strong relationship between the presence of Sycamore maple trees (*Acer pseudoplatanus)* and the incidence of AM. Trees were either found right on the pasture or as in the case of pasture B the tree was about 50 meters away. However, HGA containing seeds were consistently found on all pastures (Fig 3).

**Fig 4:**
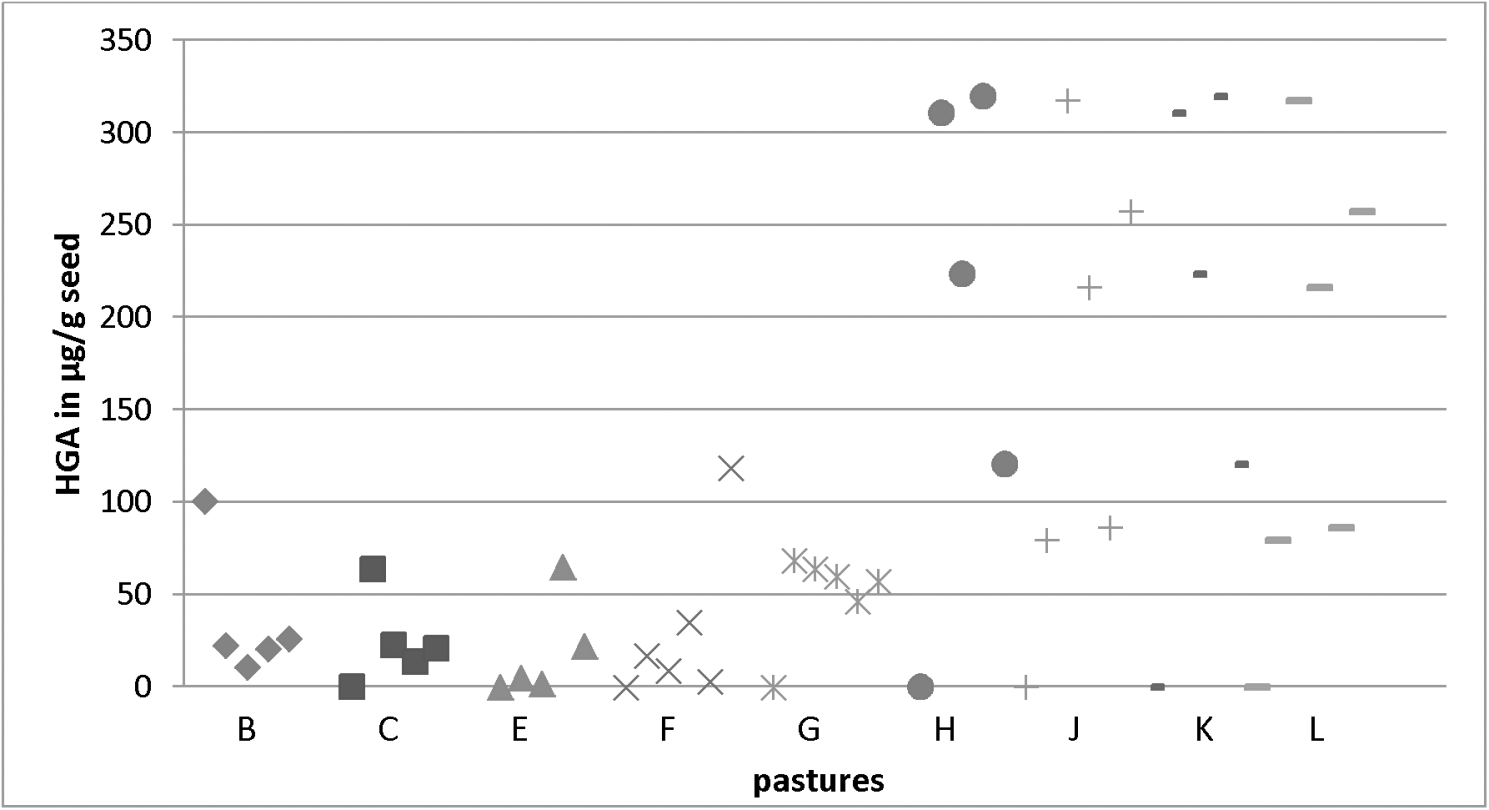
Hypoglycin A concentration in seeds of *Acer pseudoplatanus* collected from affected pastures detected by LC-ESI-MS/MS analysis. pasture B,C,E,F sampled in 2013; pasture G,H,J,K,L sampled in 2014.

Seed sampling was performed on 9 pastures with AM occurrence and the HGA concentrations of the seeds were analysed. HGA concentrations in sycamore maple seeds varied highly from seed to seed between 0.7–111.6 μg/seed. The average weight of the collected seeds was 314 mg (range from 191–428 mg). Both chosen sampling methods showed a very high variation of HGA contents (1.7–319.8 μg/g seed; Fig 4).

Body fluids of all affected horses contained the toxic amino acid HGA (Table 3). Measured concentrations in serum ranged from 387.8–8,493.8 μg/L and in urine from 143.8–926.4 μg/L. Cograzing horses showed HGA concentrations between the controls and diseased horses, ranging from 37.8 and 328.5 μg/L (n = 12; p < 0.01,) in serum, and between 18.9 and 35.1 μg/L (n = 4; p < 0.01,) in urine. HGA content in serum and urine of control horses (no access to AM-pastures) was below the limit of detection (< 10 μg/L). The results with statistical analysis are summarized in Table 4, wherein significance is indicated by p-values < 0.05.

**Table 3:** Measured concentrations of HGA in serum and urine of affected and cograzing horses.

Horse group and No.		HGA [μg/L]	
		serum	urine
AH	A 1	387.8	157.9
	A 2	468.1	*
	B 1	1734.3	143.8
	C 4	878.4	*
	D 1	4032.6	531.6
	E 1	2058.6	338.4
	G 1	8493.8	191.8
	H 1	4014.3	926.4
CG	J 2	328.5	30.8
	J 3	95.3	22.8
	J 4	197.5	35.1
	J 5	81.8	18.9
	K 2	76.8	*
	K 3	56.5	*
	K 4	46.4	*
	K 5	84.6	*
	L 2	37.8	*
	L 3	66.7	*
	L 4	66.9	*
	L 5	166.4	*

AH = affected horses

CG = cograzing horses

*sample not available.

**Table 4:** Statistical analysis according to Dunn’s multiple comparison procedure within the measured HGA parameters.

HGA in μg/L	Kruskal-Wallis test p-value	group comparison	Dunn‘s test p-value
urine	0.0038	AH vs. CG	<0.0001
		AH vs. controls	<0.0001
		controls vs. CG	>0.05
serum	<0.0001	AH vs.CG	0.00734
		AH vs. controls	<0.0001
		controls vs. CG	>0.05

AH = affected horses

CG = cograzing horses

significance is indicated by p-values < 0.05, results show that the groups CG and controls did not differ among each other, but both groups (CG, controls) are different from AH.

### Hypoglycin A in mg HGA/horse

Referring measured serum HGA concentration to the bwt of the horses and their estimated total blood volumes revealed a minimal MTD in horse A1 with 17.47 mg HGA/horse and a maximal MTD in horse G1 with 128.68 mg HGA/horse (Table 5). Within the set of cograzing horses the mean of the determined HGA concentrations was used for comparison. Cograzing horses showed HGA concentrations between the controls and diseased horses with a mean of 5.99 mg HGA/horse, thereby in close proximity to the minimal MTD of horse A1. The HGA content in serum of control horses was calculated with the detection line (< 10 μg HGA/ L) and revealed amounts of < 0.5 mg HGA/horse.

**Table 5:** HGA concentration in mg HGA/horse subjected to the blood volumes of the horsesHGA.

HGA	AH								CG	Controls
	A1	A2	B1	C4	D1	E1	G1	H1		
[μg/L]serum	387.8	468.1	1734.3	878.4	4032.6	2058.6	8493.8	4014.3	108*	< 10
bwt in kg	446	450	368	300	350	450	150	130	550	500
BV** [L]	45.04	45.45	37.17	30.30	35.35	45.45	15.15	13.13	55.55	50.50
[mg]/horse	17.47	21.28	64.46	26.62	142.55	93.56	128.68	52.71	5.99	< 0.5

AH = affected horses

CG = cograzing horses

*mean of CG (n = 12)

**BV = total blood volume: calculated with 101 ml/kg bwt [33].

## Discussion

In the present study acquired MADD was diagnosed in AM horses by increases in short and medium chain serum acylcarnitines [2], [17]. In addition to the presence of MCPA-conjugates in blood or urine of affected horses, HGA, as the possible cause for developing a MADD, was measured in the seeds of *Acer pseudoplatanus* from affected pastures and in body fluids of AM horses. To our knowledge HGA levels in body fluids of affected horses during an outbreak of AM have not been reported yet. In previous studies the presence of serum MCPA-carnitine was a consistent feature [16] and was absent in horses without contact to *Acer spp*. on pastures. In this study MCPA-carnitine concentrations in serum of affected horses were highly variable and ranged from 166–652 nmol/L. The highest concentration (4.8–102.4 nmol/L) reported by Valberg et al. [16] is within the range of our results. Votion et al. [13] measured lower concentrations of 20.4 μ 17.2 nmol/L. Since the use of the same internal standard for analysis (octanoylcarnitine) allows the comparison of the results between the studies these differences are most likely due to the amount of ingested toxic material and metabolism. The lowest concentration of MCPA-carnitine in this study was found in a horse (C4) exhibiting the lowest concentration of short and medium chain acylcarnitines (Valeryl-, Hexanoyl-, Isovaleryl-, Valeryl-, Octanoyl- and Decenoylcarnitine), but a high concentration of HGA in serum, respectively. Possibly the early euthanasia of this horse led to an immediate stop of metabolism of the toxic amino acid right after the beginning of intoxication. At this pasture C with 4 Haflinger horses, one after another developed an AM, although the horses did not have access to AM affected pasture after the first horse became ill. Within 3 days all horses died (C1-C3) or were euthanized (C4), respectively. Possibly different diet preferences of the horses on pastures increased the toxicity of HGA. Previous studies revealed different amounts of HGA as an acute toxic dose (130–150 mg HGA/kg bwt [36], 100 mg HGA/kg bwt [37]) or as the MTD (1.5 mg HGA/kg bwt [38]) leading to the conclusion that the susceptibility of animals to HGA is influenced by carbohydrate/protein ratio in their diet or the composition of the administered hypoglycin preparation.

Concentrations of HGA in seeds of *Acer pseudoplatanus* from AM pastures ranging from 0.7 to 111.6 μg/seed are similar to previously reported values with 4–56 μg/seed [16] and 3.6–252.9 μg/seed [27]. The latter showed high variation of HGA content in individual seeds collected from a single tree. Even though pooled samples (five seeds, triplicated samples) were used, the interpretation of the results remains difficult. A direct link between measured HGA amounts in seeds and the number of affected horses cannot be drawn. Farms without reported AM cases showed the highest concentrations of HGA in the collected seeds from these pastures [27].

The level of HGA in affected horses on AM-pastures calculated with their bwt and the estimated total blood volume is similar to the MTD in rats as determined by Blake et al. [38] using ackee as the source. Valberg et al. [16] converted the MTD for rats to a MTD for horses by using body surface area and the equation 1.5 mg HGA/kg bwt * bwt ^0.75^/6 (rat km value) and determined a MTD of 26.5 mg HGA/500 kg horse. This calculation can be confirmed by our results because the level of HGA in the present study ranged from 17.47 mg HGA/horse and 128.68 mg HGA/horse and especially the minimal dosage is in the field of the calculation of Valberg et al. [16]. Extrapolating 26.5 mg HGA/horse on the highest measured HGA concentration (820.8 μg/seed, [27]) 32 seeds would be sufficient to poison a 500 kg horse. Especially in autumn, when the first seeds drop to the ground, for example after a windstorm, the percentage of seeds with high HGA content available on the surface of the pasture is very high, and horses would probably not hesitate or even would not notice the ingestion of a limited number of seeds. However, to evaluate the HGA concentrations in the horse it is important to also look at the measured concentrations of the MCPA-conjugates. Since HGA is rapidly metabolized to MCPA, both values comprise essential information about HGA ingestion. Thus, looking at only body fluid HGA levels possibly underestimates the amount of HGA ingested by the horse.

Valberg et al. [16] included horses without clinical symptoms cograzing on the same pasture as an “ideal” control group. However, these horses incorporate HGA, although at lower concentrations (~5.99 mg HGA/horse, Table 5). The ingestion of seeds of *Acer pseudoplatanus* from cograzing horses on pastures was demonstrated [39]. Since the seeds have no characteristics which make them unpalatable to horses, there is a chance that they will be consumed during grazing. If these consumed seeds contain a high concentration of HGA, it could result in poisoning [40]. An ideal control group consists of horses without access to seeds of *Acer spp*. This distinction is necessary because both groups are clinically unaffected, but the likelihood of developing an AM-disease is higher within the cograzing horses because of detectable concentrations of HGA and MCPA-conjugates.

Currently we are unable to standardise the MTD of HGA for horses, but suggestions about possible quantities can be deduced based on our results. Concentrations of HGA in cograzing horses were higher than in controls but always lower compared to affected horses, although some of these horses (Table 3) showed HGA-contents in close proximity to affected horses. This assumption leads to the conclusion that the detected HGA concentrations in horses and therefore a possible intoxication mainly depends on i) the high variation of HGA levels in seeds and thus ii) the availability of highly toxic seeds and iii) the different feed preferences of the individual horse as well as iv) the time of blood sampling (before or after a peak). Obviously, cograzing horses as well as affected horses ingested HGA containing material (most likely *Acer pseudoplatanus* seeds, [39]), but the cograzing group did not acquire the necessary toxic amount of HGA at the time of sampling. The detection of low but elevated HGA concentration in blood samples of cograzing horses should result in a complete closing off the pasture to all horses. Still, clinical symptoms may develop shortly afterwards, but an early initiated therapy may decelerate the metabolism of HGA to the toxic MCPA-conjugates and prognosis could be improved.

## Conclusions

This study correlated an outbreak of AM with high contents of HGA in seeds of *Acer pseudoplatanus* on affected pastures and in body fluids of diseased horses. Although the horses in our study obtained additional foodstuffs (hay, mixed feeds) and had in most cases only limited access to the pasture, they ingested enough HGA to be poisoned. The recommendation of preventing horses from grazing on affected pastures with *Acer pseudoplatanus* is indispensable, however, the analysis of HGA in horses grazing on pastures with *Acer pseudoplatanus* without reported cases allows a quick survey, whether or not HGA was ingested. We suggest that the availability of a test for early detection of HGA in clinically normal horses might represent a valuable diagnostic tool for prophylaxis, providing information on the HGA-status of horses at risk, similar to a diagnostic marker.

The causal chain of HGA availability by *Acer pseudoplatanus* seeds, HGA ingestion and HGA as well as toxic metabolites in body fluids of affected horses, was further substantiated by our study. Although sycamore maple seeds were the likely source of toxin in all horses in the present study other related trees or plants as a cause for the disease cannot be excluded.

## Supporting Information

S1 Table MS parameters for MRM-transitions. (DOCX)

## References

[1] van Galen G, Marcillaud Pitel C, Saegerman C, Patarin F, Amory H, Baily JD et al (2012a) European outbreaks of atypical myopathy in grazing equids (2006–2009): Spatiotemporal distribution, history and clinical features. Equine Vet J 44:614 22448904 10.1111/j.2042-3306.2012.00556.x

[2] Sponseller BT, Valberg SJ, Schultz NE, Bedford H, Wong E, Kersh K et al (2012) Equine multiple acyl-CoA dehydrogenase deficiency (MADD) associated with seasonal pasture myopathy in the midwestern United States. J Vet Intern Med 26: 1012–1018 10.1111/j.1939-1676.2012.00957.x 22708588

[3] Votion DM, Delguste C, Baise E, Cassart D, Desmecht D, Linden A et al (2003) Diagnostic differentielen cas de presomption de myopathie atypique des equides: illustrationau travers de cas referesa la Faculte de Medecine Veterinaire del’Universite de Liege au cours du printemps. Annales de Medecine Veterinaire 147:183–193

[4] Votion DM, Linden A, Saegerman C, Engels P, Erpicum M, Thiry E et al (2007) History and clinical features of atypical myopathy in horses in Belgium (2000–2005). J Vet Intern Med 21:1380 18196750 10.1892/07-053.1

[5] Palencia P and Rivero JL (2007) Atypical myopathy in two grazing horses in northern Spain. Vet Rec 161: 346–348 17827475 10.1136/vr.161.10.346

[6] Bowen JN, Craig JF (1942) Myoglobinuria in horses. Vet Rec 35:354

[7] Hosie BD, Gould PW, Hunter AR, Low JC, Munro R, Wilson HC. (1986) Acute myopathy in horses at grass in east and south east Scotland.Vet Rec 119: 444–449 3798693 10.1136/vr.119.18.444

[8] Whitwell KE, Harris P and Farrington PG (1988) Atypical myoglobinuria: acute myopathy in grazing horses. Equine Vet J 20: 357–363 3053157 10.1111/j.2042-3306.1988.tb01545.x

[9] Harris P and Whitwell K. (1990) Atypical myoglobinuria alert. Veterinary 127:603 2075692

[10] Hillam RA (1991) Atypical myoglobinuria. The Veterinary Record 128:166 10.1136/vr.128.7.166-a 2028581

[11] Finno CJ, Valberg SJ, Wuenschmann A, Murphy MJ (2006) Seasonal pasture myopathy in horses in the midwestern United States: 14 cases (1998–2005). J Am Vet Med Assoc 229:1134–1141 17014363 10.2460/javma.229.7.1134

[12] Brandt K, Hinrichs U, Glitz F, Landes E, Schulze C (1997) Atypische Myoglobinurie der Weidepferde. Pferdeheilkunde 13: 27–34

[13] Votion DM, van Galen G, Sweetman L, Boemer F, de Tullio P, Dopagne C et al (2014) Identification of methylenecyclopropyl acetic acid in serum of European horses with atypical myopathy. Equine Vet J 2: 146–149 10.1111/evj.12117 23773055

[14] Votion DM, Serteyn D (2008) Equine atypical myopathy: a review; Veterinary J., 178:185–190 10.1016/j.tvjl.2008.02.004 18375157

[15] van Galen G, Saegerman C, Marcillaud Pitel C, Patarin F, Amory H, Baily JD et al (2012b) European outbreaks of atypical myopathy in grazing horses (2006–2009): Determination of indicators for risk and prognostic factors. Equine Vet J 44:621 22413891 10.1111/j.2042-3306.2012.00555.x

[16] Valberg SJ, Sponseller BT, Hegeman AD, Earing J, Bender JB, Martinson KL et al (2013): Seasonal pasture myopathy/atypical myopathy in North America associated with ingestion of hypoglycin A within seeds of the box elder tree. Equine Vet J 45:419 10.1111/j.2042-3306.2012.00684.x 23167695

[17] Westermann CM, Dorland L, Votion DM, de Sain-van der Velden MGM, Wijnberg ID, Wanders RJA et al (2008) Acquired multiple Acyl-CoA dehydrogenase deficiency in 10 horses with atypical myopathy. Neuromuscul Disord 18: 355 10.1016/j.nmd.2008.02.007 18406615

[18] Frerman FE and Goodman SI (1985) Deficiency of electron transfer flavoprotein or electron transfer flavoprotein: ubiquinone oxidoreductase in glutaric acidemia type II fibroblasts. Proc Natl Acad Sci U.S.A. 82:4517–4520 2989828 10.1073/pnas.82.13.4517 PMC391133

[19] Meda HA, Diallo B, Buchet JP, Lison D, Barennes H, Amadou Ouangre PhD et al (1999) Epidemic of fatal encephalopathy in preschool children in Burkina Faso and consumption of unripe ackee (Blighia sapida) fruit. Lancet 353:536–540 10028981 10.1016/S0140-6736(99)01088-0

[20] Joskow R, Belson M, Vesper H, Backer L, Rubin C (1999) Ackee fruit poisoning: an outbreak investigation in Haiti 2000–2001, and review of the literature. Clinical Toxicology 44:267–3 10.1080/15563650600584410 16749544

[21] Cassart D, Baise E, Cherel Y, Delguste C, Antoine N, Votion D et al (2007) Morphological alterations in oxidative muscles and mitochondrial structure associated with equine atypical myopathy. Equine Vet J 39:26–32. 17228591 10.2746/042516407x157765

[22] Verheyen T, Decloedt A, De Clercq D and van Loon G (2012) Cardiac changes in horses with atypical myopathy. J Vet Intern Med.26: 1019–1026 10.1111/j.1939-1676.2012.00945.x 22646196

[23] Delguste C, Cassart D, Baise E, Linden A, Schwarzwald C, Feige K. et al (2002) Myopathies atypiques chez leschevaux au pré: une série de cas en Belgique. Ann. Med. Vet. 146: 231–243.

[24] Chase GW Jr, Landen WO Jr, Soliman AG (1990) Hypoglycin A content in the aril, seeds, and husks of ackee fruit at various stages of ripeness. J Association of Official Analytical Chemists 73(2): 318–319 2324040

[25] Bowen-Forbes CS, Minott DA (2011) Tracking hypoglycins A and B over different maturity stages: implications for detoxification of ackee (Blighia sapida K.D. Koenig) fruits: Journal of Agricultural and Food Chemistry 59(8): 3869–3875 10.1021/jf104623c 21410289

[26] van der Kolk JH, Wijnberg ID, Westermann CM, Dorland L, de Sein-van der Velden MGM, Kranenburg LC et al (2010) Equine acquired multiple acyl-CoA dehydrogenase deficiency (MADD) in 14 horses associated with ingestion of Maple leaves (Acer pseudoplatanus) covered with European tar spot (Rhytisma acerinum). Mol Genet Metab 101:28 10.1016/j.ymgme.2010.06.019 20655779

[27] Unger L, Nicholson A, Jewitt E.M, Gerber V, Hegemann A, Sweetmann L et al(2014) Hypoglycin A Concentrations in seeds of Acer pseudoplatanus trees growing on atypical myopathy-affected and control pastures. J Vet Intern Med 28(4): 1289–1293 10.1111/jvim.12367 24863395 PMC4857957

[28] Carlier J, Guitton J, Moreau C, Boyer B, Bevalot F, Fanton L et al (2015). A validated method for quantifying hypoglycin A in whole blood by UHPLC- HRMS/MS. J of Chromatography B. 978–979: 70–77 10.1016/j.jchromb.2014.11.029 25531872

[29] Bressler R, Corredor C, Brendel K (1969) Hypoglycin and hypoglycin-like compounds. Pharmacological Reviews (21) No. 2:105–130 4897145

[30] Fincham AG (1977)The determination of hypoglycin-A in blood and plasma. West Indian Med. J 26: 62 878454

[31] Sander J, Cavalleri JM, Terhardt M, Bochnia M, Zeyner A et al (2015, submitted) Rapid diagnosis of hypoglycin A intoxication in atypical myopathy of horses. 10.1177/1040638715624736 26965229

[32] Ziegler J, Abel S (2014) Analysis of amino acids by HPLC/electrospray negative ion tandem mass spectrometry using 9-fluorenylmethoxycarbonyl chloride (Fmoc-Cl) derivatization, Amino Acids 46: 2799 10.1007/s00726-014-1837-5 25218137

[33] Zeller R und Siewert R (1964) Blutvolumenuntersuchungen beim Pferd mit Jod^131^ HSA (RIHSA), Zentralblatt für Veterinärmedizin Reihe A, 11 Issue 6: 523–537

[34] Elliott AC and Hynan LS(2010) Macro implementation of a multiple comparison post hoc test for a Kruskal–Wallis analysis: computer methods and programs in biomedicine. 102:75–80 10.1016/j.cmpb.2010.11.002 21146248

[35] Koeller G, Gieseler T, Schusser GF (2014) Hematology and serum biochemistry reference ranges of horses of different breeds and age measured with newest clinicopathological methods. Pferdeheilkunde 30(4): 381–393.

[36] Feng PC and Kean EA (1955) Influence of diet on the acute toxicity of hypoglycin A in rats. Brit J Nutr 9: 368 13284218 10.1079/bjn19550052

[37] Feng PC and Patrick SJ (1958) Studies of the action of hypoglycin-A, an hypoglycaemic substance. Br J Pharmacol. Chemother 13: 125 10.1111/j.1476-5381.1958.tb00206.x PMC1481712 13536275

[38] Blake OA, Bennink MR and Jackson JC (2006) Ackee (Blighia sapida) hypoglycin A toxicity: dose response assessment in laboratory rats. Food Chem Toxicol 44: 207–213 16099087 10.1016/j.fct.2005.07.002

[39] Aboling S, Schliephacke A, Mueller JM, Kamphues J (2015a) Nachweis von Berg-Ahorn im Magen-Darminhalt eines Pferdes mit Verdacht auf Atypische Myopathie. Pferdeheilkunde 31:135–139

[40] Aboling S, Cavalleri JMV, Cehak A, Johnson J, Ziegler J, Sander J et al (2015b) Aspects of nutritional ecology including hypoglycin A concentration in various organs of the genus maple (Acer)-relevance for the sickness atypical myopathy in horses. Proc of the Soc of Nutr Phys 24:124

