# Supplementary material for: Hypoglycin A Content in Blood and Urine Discriminates Horses with Atypical Myopathy from Clinically Normal Horses Grazing on the Same Pasture
Source: PLoS One. 2015 Sep 17;10(9):e0136785. doi: 10.1371/journal.pone.0136785 (PMC4574941; doi:10.1371/journal.pone.0136785)
Supplement: S1 Table — (DOCX) [file pone.0136785.s001.docx]

**Supporting Information**

| S1 Table: MS parameters for MRM-transitions   \| Analyte \| MRM transitions \| Declustering potential (DP), V \| Entrance potential (EP), V \| Cell entrance potential (CEP), V \| \| --- \| --- \| --- \| --- \| --- \| \| Fmoc-Hypoglycin \| **362→166**  *362→122^a^* \| -30  -30 \| -3  -3 \| -20  -20 \| \| Fmoc-Norvaline \| **338→116**  *338→14^a^* \| -15  -15 \| -8  -8 \| -25  -25 \| | |
| --- | --- | --- | --- | --- | --- | --- | --- | --- | --- | --- | --- | --- | --- | --- | --- | --- |
| Quantifier and qualifier transitions are indicated in bold and italics, respectively |  |
